# Efficacy and safety of belimumab combined with the standard regimen in treating children with lupus nephritis

**DOI:** 10.1007/s00431-024-05662-9

**Published:** 2024-06-28

**Authors:** Huarong Li, Chaoying Chen, Hongxian Yang, Juan Tu

**Affiliations:** Department of Nephrology, Children’s Hospital Affiliated to Capital Institute of Pediatrics, Beijing, 100020 China

**Keywords:** Belimumab, Children, Efficacy, Lupus nephritis, Safety

## Abstract

**Abstract:**

The purpose of this study is to evaluate the efficacy and safety of belimumab combined with the standard regimen in treating children with active lupus nephritis. This single-center, retrospective cohort study used clinical data of children with newly active lupus nephritis hospitalized in the Department of Nephrology between December 2004 and February 2023. Patients were divided into a belimumab or traditional treatment group according to whether or not they received belimumab. Renal remission and recurrence rates and glucocorticoid dose were compared between groups. Forty-seven children (median age 11 years) were enrolled, including 30 and 17 children in the traditional treatment and belimumab groups, respectively. The Systemic Lupus Erythematosus Disease Activity Index-2000 (SLEDAI-2000) score of children in the belimumab group (23.59 ± 7.78) was higher than that in the traditional treatment group (19.13 ± 6.10) (*P* = 0.035). The two groups showed no significant difference in the frequency of pyuria, gross hematuria, and the levels of 24-h proteinuria and estimated glomerular filtration rate. The complement C3/C4 in the belimumab group recovered faster than that in the traditional treatment group (*P* < 0.05). There were no between-group differences in the complete renal remission rate at 6 or 12 months (*P* = 0.442, *P* = 0.759). There were no between-group differences in 1-year recurrence rate (*P* = 0.303). Furthermore, 6 and 12 months after treatment, glucocorticoid doses were lower in the belimumab than the traditional treatment group (17.87 ± 6.96 mg/d vs. 27.33 ± 8.40 mg/d, *P* = 0.000; 10.00 (5.3) mg/d vs. 13.75 (10.0) mg/d, *P* = 0.007), respectively.

***Conclusion*:**

With an equivalent renal remission rate, belimumab combined with the standard traditional regimen might promote the tapering of glucocorticoids, and the incidence of adverse events is low.

**Table:** 

**What is known**:
• *Belimumab is documented as an adjunctive treatment with systemic lupus erythematosus (c-SLE) LN with efficacy.* • *Due to the paucity of studies, its effects and side effects in children with LN remain unclear.*
**What is new**:
• *This single-center, retrospective cohort study evaluated the efficacy and safety of belimumab combined with the standard regimen in treating children with proliferative LN.* • *Belimumab combined with the standard traditional treatment might promote the tapering of glucocorticoids, while exhibiting a low occurrence of adverse events.*

**Supplementary Information:**

The online version contains supplementary material available at 10.1007/s00431-024-05662-9.

## Introduction

Systemic lupus erythematosus (SLE) is an autoimmune disease that invades multiple organs and systems. Childhood-onset SLE accounts for only 15–20% of all SLE cases [1]; however, it is more acute, affects more systems, has a more active course, and has a worse prognosis than adult SLE, especially in patients with kidney involvement. Consequently, over 50% of children with SLE develop lupus nephritis (LN) [2], usually within the first 2 years of diagnosis [3]. The standard induction regimen of corticosteroids combined with immunosuppressants, including cyclophosphamide (CTX) or mycophenolate mofetil (MMF), has improved the renal prognosis; however, over one-third of children may still experience recurrence after remission [2].

Belimumab can bind to B cell-activating factor (BAFF) (also known as B lymphocyte stimulator, Blys) with high affinity. It blocks Blys from binding to receptors on B cells and inhibits B cell proliferation and plasma cell differentiation, thereby reducing the production of autoantibodies in the serum and thus treating SLE [4]. As the first biologic approved for SLE treatment in children aged ≥ 5, it was also approved on February 10, 2022, to be combined with traditional therapy for active LN in adults. However, research on the clinical application of belimumab in children with LN is limited. Therefore, we collected clinical data of children with active LN who received belimumab combined with standard traditional regimens at our center. We aimed to evaluate the efficacy and safety of belimumab in this population.

## Materials and method

### Research participants

This single-center, retrospective cohort study collected the clinical data of newly diagnosed patients with active LN hospitalized in the nephrology department between December 2004 and February 2023. This study was performed in line with the principles of the Declaration of Helsinki.

### Inclusion and exclusion criteria

1. The following are the inclusion criteria: All enrolled children met the 2019 European League Against Rheumatism/American College of Rheumatology classification criteria for lupus nephritis [5], and all cases diagnosed before 2016 were reevaluated according to these criteria.
2. The following are the exclusion criteria: (1) children with LN who had been regularly treated with immunosuppressants and biologics within the past 3 months and (2) LN was already in remission at the onset of belimumab.

### Grouping

The participants were divided into the belimumab and traditional treatment groups based on whether they received belimumab treatment or not. All participants chose their treatment based on personal preference and signed informed consent.

1. Traditional treatment group: All patients were administered glucocorticoids or immunosuppressants. The immunosuppressants included CTX, MMF, cyclosporine A, and tacrolimus. Glucocorticoid tapering and dosing were based on the treating physician and clinical response over time.
2. Belimumab group: On the basis of traditional treatment, we added a belimumab treatment regimen as an intravenous infusion of 10 mg/kg at weeks 0, 2, and 4, followed by another intravenous infusion of 10 mg/kg every 4 weeks for a maximum duration of 52 weeks.

### Observation and evaluation indicators

1. Data on sex, age, LN course, clinical and pathological classification, and extrarenal-affected organs of children in both groups at the time of enrollment and follow-up were collected through outpatient follow-up visits and readmission medical records or phone calls after discharge.
2. Observation indicators: All enrolled patients had regular follow-ups and no data were imputed. Laboratory indicators of patients at 0, 6, and 12 months after treatment included 24-h proteinuria and estimated glomerular filtration rate (eGFR) based on the Schwartz formula.
3. Effectiveness evaluation: Indicators such as renal remission rate, recurrence rate, Systemic Lupus Erythematosus Disease Activity Index-2000 (SLEDAI-2000) score, and glucocorticoid dosage at 6 and 12 months of treatment were evaluated.
4. Safety evaluation: During the follow-up period of belimumab treatment, adverse events, including infusion reactions, hypersensitivity reactions, infections, and blood immunoglobulin levels, were recorded.

### Treatment response [6]

The following criteria were adopted to define treatment response:

1. Complete remission (CR): normal renal function (eGFR > 90 mL/min/1.73 m^2^), 24-h proteinuria < 0.5 g/d, or urine protein: creatinine ratio (UPCR) < 0.5 mg/mg.
2. Partial remission (PR): renal function is stable, and proteinuria is reduced by > 50% from baseline.
3. No renal remission (NR): failure to achieve partial or complete remission within 6–12 months of therapy.
4. Renal recurrence: In patients with complete renal remission, proteinuria increased by > 50%, and/or eGFR decreased by > 25% from baseline.

### Statistical methods

SPSS 26 software (IBM©, Chicago, IL, USA) was used for statistical analysis. Kolmogorov–Smirnov test was used to evaluate the normal distribution of continuous variables. Quantitative parametric data were presented as mean and standard deviation (mean ± SD) and were analyzed by a *t*-test. Quantitative nonparametric data are presented as median and interquartile range (median IQR) and were analyzed by the Mann–Whitney test. A multivariate Cox regression model was used to evaluate the renal survival (time of partial and complete remission), and relevant variables that were significantly associated with the renal survival by univariate analysis were included in multivariate models. Statistical significance was set at *P* < 0.05.

## Results

### General information

Forty-seven cases were enrolled, with a median age of 11.0 years (9.0, 12.0 years), including 18 males (38.3%) and 29 females (61.7%). The belimumab group had 17 cases, with a median age of 11.0 years (9.0, 13.0 years), including 5 males (29.4%) and 12 females (70.6%). The initial diagnoses were made between September 2020 and February 2023. In the traditional treatment group, there were 30 cases with a median age of 10.5 years (8.75, 12.0 years), including 13 males (43.3%) and 17 females (56.7%). The initial diagnosis was between December 2004 and April 2022. During September 2020 and February, only one case chose traditional treatment.

### Clinical manifestations and treatment

1. Extrarenal manifestations: At disease onset, there were no statistically significant differences between the two groups regarding the extrarenal organs involved including mucocutaneous, musculoskeletal, hematologic, and nervous systems. The SLEDAI-2000 score in the belimumab group (23.59 ± 7.78) was higher than that in the traditional treatment group (19.13 ± 6.10), with statistical significance (*t* = 2.176, *P* = 0.035).
2. Renal changes: The two groups had no significant differences in the frequency of pyuria, gross hematuria, and the levels of 24-h proteinuria and eGFR. Among all cases, acute nephritis (16/47, 34.0%) and nephrotic syndrome (23/47, 48.9%) were the most common, and there was no statistically significant difference in clinical subtype distribution between both groups (*χ*^2^ = 2.192, *P* = 0.533). Forty-two patients underwent renal tissue biopsy, of which 41 (97.6%) were type III (including type III + V) and type IV (including type IV + V), with type IV being the most subtype. There was no significant difference in pathological subtype distribution between both groups (*χ*^2^ = 1.674, *P* = 0.643). Although the average activity index (AI) of the belimumab group (10.47 ± 2.72) was higher than that of the traditional treatment group (9.59 ± 2.78), there was no statistically significant difference (*t* = 0.935, *P* = 0.357), and the median chronic index(CI) between both groups was not statistically different (*Z* = 1.244, *P* = 0.322) (Table 1).

**Table 1:** Comparison of baseline clinical and laboratory data between the traditional treatment group and the belimumab group

	Traditional treatment group (*n* = 30)	Belimumab group (*n* = 17)	*t*/*Z*/*χ*^2^ value	*P*
Male (*n*, %)	13 (43.3)	5 (29.4)	0.89	0.345
Age (year) Median (IQR)	10.5 (8.75, 12.0)	11.0 (9.0, 13.0)	− 0.989	0.328
Organ involvement				
Mucocutaneous (*n*, %)	14 (46.7)	9 (52.9)	0.171	0.679
musculoskeletal (*n*, %)	6 (20.0)	4 (23.5)	0.008	0.931
Hematologic (*n*, %)	24 (80.0)	17 (100.0)	2.309	0.129
Nervous system (*n*, %)	6 (20.0)	3 (17.7)	0.036	0.85
Kidney				
Gross hematuria (*n*, %)	14 (46.7)	7 (41.2)	0.132	0.716
Pyuria (*n*, %)	21 (70.0)	13 (76.5)	0.061	0.805
24-h proteinuria (mg/kg·d) Median (IQR)	63.3 (22.3, 146.2)	75.9 (34.5, 117.0)	− 0.133	0.894
Scr (μmol/L) Median (IQR)	62.54 (48.5, 74.6)	65.50 (49.5, 102.4)	− 0.831	0.406
eGFR (mL/min·1.73 m^2^) Mean ± SD	94.72 ± 32.07	81.02 ± 37.88	1.298	0.201
Clinical typing (*n*, %)			2.192	0.533
Hematuria-proteinuria type	3 (10.0)	2 (11.8)		
Acute nephritis type	12 (40.0)	4 (23.5)		
Nephrotic syndrome type	14 (46.7)	9 (52.9)		
Acute progressive nephritis type	1 (3.33)	2 (11.8)		
Pathological classification (*n*, %)			1.674	0.643
II	1 (4.0)	0 (0.0)		
III	1 (4.0)	0 (0.0)		
IV	19(76.0)	15 (88.2)		
III/IV + V	4(16.0)	2(11.8)		
AI Mean ± SD	9.59 ± 2.78	10.47 ± 2.72	− 0.935	0.357
CI Median (IQR)	0.0 (0.0, 1.5)	0.0 (0.0, 0.0)	− 1.244	0.322
C3 (g/L) Median (IQR)	0.27 (0.2, 0.5)	0.32 (0.2, 0.4)	− 0.444	0.657
C4 (g/L) Median (IQR)	0.04 (0.0, 0.1)	0.06 (0.0, 0.1)	− 0.692	0.489
SLEDAI-2000 Mean ± SD	19.13 ± 6.10	23.59 ± 7.78	− 2.176	0.035

*Scr* serum creatinine, *eGFR* estimated glomerular filtration rate, *SLEDAI-2000* Systemic Lupus Erythematosus Disease Activity Index-2000

### Immune induction therapy

Among the two groups, CTX was the most commonly used immunosuppressive induction therapy with 39 cases. Among them, the traditional treatment and belimumab groups had 27 cases (93.10%) and 12 cases (70.59%), respectively, and there was no difference in treatment between the two groups (Table 2).

**Table 2:** Comparison of induction therapy between the traditional treatment and belimumab groups

	Traditional treatment group (*n* = 30)	Belimumab group (*n* = 17)	*χ*^2^	*P*
Immune induction therapy, *n* (%)			2.647	0.104
GC + CTX	27 (90.0)	12 (70.6)		
GC + MMF	2 (6.7)	5 (29.4)		
CNI, *n* (%)	4 (13.33)	6 (35.29)	1.951	0.163
HCQ, *n* (%)	16 (53.33)	8 (47.06)	0.171	0.679
PE, *n* (%)	0(0.0)	1(5.9)		0.362

*GC* glucocorticoid, *CTX* cyclophosphamide, *MMF* mycophenolate mofetil, *CNI* calcineurin inhibitors, *HCQ* hydroxychloroquine, *PE* plasma exchange

### Belimumab treatment

Seventeen enrolled cases of LN received belimumab therapy from 2 weeks to 3 months after starting traditional therapy with an average of 1.9 ± 1.4 months. Fourteen patients completed 52 weeks of treatment, whereas the remaining three cases received belimumab for 12–20 weeks, due to continuous renal remission and refused to continue use.

### Validity

**Fig. 1:**
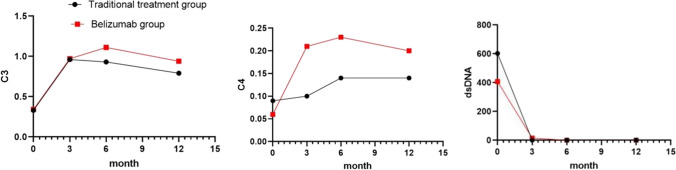
Changes in complement C3/C4/anti dsDNA antibodies in the traditional treatment and belimumab groups. dsDNA, anti-double-stranded DNA

**Fig. 2:**
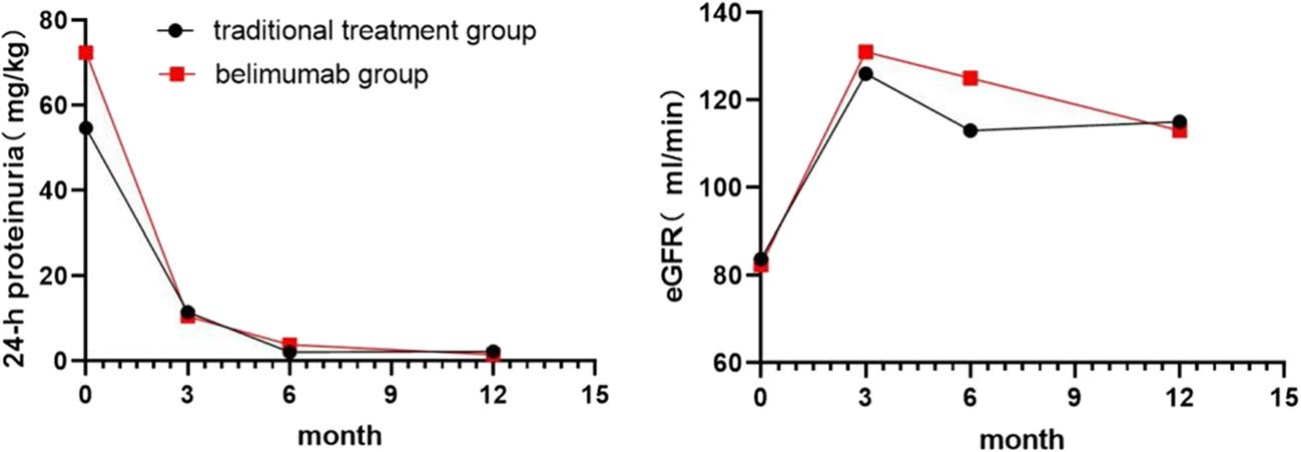
Changes in 24-h proteinuria and eGFR in the traditional treatment group and belimumab group. eGFR, estimated glomerular filtration rate

1. Activity: The SLEDAI-2000 score was comparable between the two groups at 6 months of treatment, whereas at 12 months, the belimumab group had a lower score; however, the difference between the groups was not statistically significant. Comparing the complement C3 and C4 levels, the belimumab group recovered faster than the traditional treatment group at 3, 6, and 12 months of treatment (*P* < 0.05). There were no differences in the titer changes of anti-double-stranded DNA antibodies (dsDNA) between the two groups (Fig. 1).
2. There were no significant differences in 24-h proteinuria and eGFR between the two groups after 3, 6, and 12 months of treatment (*P* > 0.05) (Fig. 2).
3. Renal response rate: At 6 months of treatment, the CR rate of all patients was 37/47 (78.7%), with that in the belimumab group (88.2%) being higher than that in the traditional treatment group (73.3%). The PR and NR rates were lower in the belimumab group than in the traditional treatment group; however, the difference was not statistically significant. At 12 months of treatment, the CR rate of all patients was 40/44 (90.9%), and there was no significant difference in the CR or PR rates between the two groups. There were no nonresponders in either group.

#### Renal recurrence and chronic kidney disease (CKD)

In the median follow-up of 13.0 months (9.0, 28.0 months) in the belimumab group, no clinical recurrence cases were observed. At 12 months of treatment, there were four cases (13.3%) of recurrence in the traditional treatment group, all of whom were followed up on time and treated regularly (*χ*^2^ = 1.061, *P* = 0.303). As of October 31, 2023, the median follow-up time for all children in the traditional treatment group was 49.5 months (16.5, 70.0 months), of which 13 cases (43.3%) had 21 relapses all within 5 years. The median time from onset to the first recurrence was 2 years (1, 3.5 years). Ten patients (76.9%) had one relapse, one (7.7%) had two relapses, one (7.7%) had three relapses, and one (7.7%) had six relapses. Thirteen of the 21 relapses (61.9%) occurred after infection, fatigue, and irregular medication use, whereas eight (38.1%) had no triggers. At the end of follow-up, 16 patients in the traditional treatment group had become adults, of which one patient had developed stage 2 of CKD (followed up for 116 months, with six relapses), whereas the other patients showed continuous CR. Further details are presented in Table 3. After adjusting the variables including SLEDAI-2000, Scr, eGFR, and complement C3, multivariate Cox regression analysis showed a significant correlation between 24-h proteinuria and CI score and renal prognosis (OR 2.301, 95% CI 1.311–4.038, *P* = 0.004; OR 0.018, 95% CI 0.001–0.393, *P* = 0.011, respectively) (Table 4).

**Table 3:** Comparison of SLE activity and renal response rates between the traditional treatment and the belimumab groups at 6 and 12 months of treatment

	6 months				12 months			
	Traditional treatment group	Belimumab group	*t*/*Z*/*χ*^2^	*P*	Traditional treatment group	Belimumab group	*t*/*Z*/*χ*^2^	*P*
SLEDAI-2000 Median (IQR)	4 (2, 8)	4 (2, 7)	− 0.255	0.799	4 (1, 6)	0(0, 4)	− 1.507	0.132
eGFR (mL/min·1.73 m^2^) Mean ± SD	128.65 ± 26.33	121.72 ± 21.96	0.863	0.394	116.80 ± 20.90	115.36 ± 18.32	0.196	0.846
Renal remission rate, *n* (%)			1.631	0.442			0.094	0.759
CR	22 (73.3)	15 (88.2)			27 (90.0)	13 (92.9)		
PR	7 (23.3)	2 (11.8)			3 (10.0)	1 (7.1)		
NR	1 (3.3)	0 (0.0)			0 (0.0)	0 (0.0)		
Recurrence rate within 1 year, *n* (%)					4 (13.3%)	0 (0.0%)	1.061	0.303

*SLE* systemic lupus erythematosus, *CR* complete remission, *NR* no renal remission, *PR* partial remission

**Table 4:** Univariate and multivariable Cox regression analyses of belimumab effects in studied patients

Characteristics	Univariate		Multivariate	
	OR (95% CI)	*P*	OR (95% CI)	*P*
Age, per 1 year older	1.092 (0.976–1.222)	0.126	1.003 (0.999–1.006)	*0.117*
Sex, male vs female	1.708 (0.283–10.287)	0.559	1.066 (0.805–1.412)	*0.655*
Scr, per 1 μmol/L higher	1.633 (0.993–2.684)	0.043	0.993 (0.985–1.001)	0.072
eGFR, per 1 mL/min/1.73 m^2^ lower	0.976 (0.946–1.008)	0.143		
24-h proteinuria, per 1 mg/kg/d higher	1.130 (1.038–1.230)	0.005*	2.301 (1.311–4.038)	0.004*
C3, per 1 g/L higher	5.300 (0.945–29.742)	0.058		
C4, per 1 g/L higher	0.075 (0.000–623.953)	0.574		
SLEDAI-2000, per 1score higher	1.043 (0.996–1.092)	0.076		
Crescent, per 1% higher	1.023 (0.987–1.061)	0.215		
Glomerulosclerosis, per 1% higher	1.022 (0.960–1.088)	0.498		
AI, per 1 score higher	0.968 (0.706–1.328)	0.841		
CI, per 1 score higher	0.845 (0.755–0.945)	0.003*	0.018 (0.001–0.393)	0.011*

*OR* odds ratio, *CI* confidence interval

^*^P-value: significant ≤ 0.05

#### Glucocorticoid dosage

**Fig. 3:**
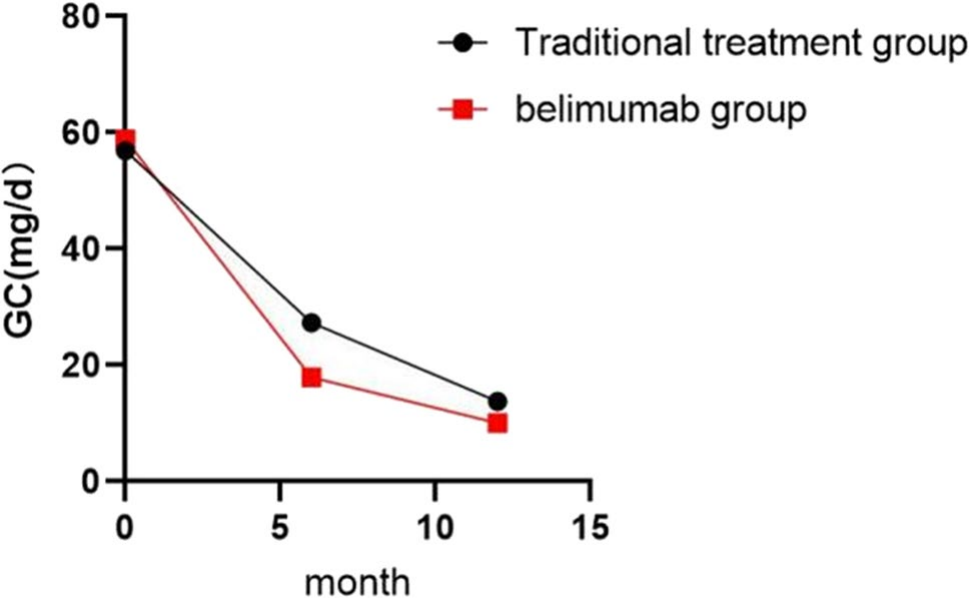
Comparison of glucocorticoid doses between the traditional treatment and belimumab groups at 6 and 12 months of treatment. GC, glucocorticoid

Both groups were treated with sufficient glucocorticoids following the guidelines [6] to induce remission. After 6 months of treatment, the glucocorticoid dosage in the belimumab group (17.87 ± 6.96 mg/d) was significantly lower than that in the traditional treatment group (27.33 ± 8.40 mg/d) (*P* < 0.001). The glucocorticoid dosage in the belimumab group (10.00 (5.3, 10.0) mg/d) after 12 months of treatment was significantly lower than that in the traditional treatment group (13.75 (10.0, 22.5) mg/d) (*P* = 0.007), with significant statistical significance. See Fig. 3 for details.

### Safety

No infusion-related reactions occurred in any patient during belimumab treatment. Nine children (52.9%) experienced acute upper respiratory tract infections two to four times, one (5.9%) had gastroenteritis, one (5.9%) had tinea versicolor, and one (5.9%) had a varicella zoster virus infection. Notably, all infections improved within 1 week, and no serious adverse reactions occurred. There was a downward trend in serum immunoglobulin M (IgM), IgG, and IgA levels compared with baseline at 6 and 12 months after belimumab treatment; however, there was no statistically significant difference (Table 5).

**Table 5:** Comparison of immunoglobulin levels in the belimumab group

	Baseline	6 months	12 months	*F*	*P*
IgG (g/L) Mean ± SD	8.65 ± 4.60	7.52 ± 3.21	7.16 ± 2.97	0.669	0.517
IgM (g/L) Mean ± SD	1.26 ± 0.47	0.95 ± 0.40	0.93 ± 0.37	2.955	0.063
IgA (g/L) Mean ± SD	0.83 ± 0.42	0.56 ± 0.30	0.64 ± 0.38	2.18	0.125

*IG* immunoglobulin

## Discussion

We evaluated the efficacy and safety of belimumab combined with the standard regimen in treating children with active lupus nephritis and found that belimumab plus standard therapy promoted rapid reduction of proteinuria, and the complement C3 and C4 levels recovered faster, although there was no difference in renal remission rate at 6 months and 1 year compared to traditional treatment alone. Our findings also demonstrate that belimumab as an adjuvant treatment can promote the tapering of glucocorticoid.

Standard treatment with corticosteroids combined with immunosuppressive agents (including CTX or MMF) improves renal prognosis, but it is not an ideal therapy. According to adult data, approximately 45% of patients with proliferative LN did not improve within 6 months of standard treatment [7], and 25–30% of patients develop end-stage kidney disease (ESKD) within 20 years [8]. A cohort study of adults with LN in the UK [9] showed that 33% of patients with histologically confirmed grade III or IV LN experienced renal recurrence at an average of 3.5 years after induction, and 44% of patients with PR experienced renal recurrence. The recurrence rate was only 5% among patients who achieved initial CR. Subsequent studies [10, 11] also suggest that an inadequate renal response to induction therapy primarily causes recurrence and poor prognosis.

Compared with adults, there are few reports on pediatric LN. In recent long-term prognostic data on pediatric LN, survival rates without advanced CKD, ESKD, or death were 92.7% and 83.2% at 10 and 20 years, respectively [12]. Proliferative LN is the most common and severe type of LN in children [13, 14] and is a risk factor for progression to CKD [15]. Recurrence is common in children, especially those aged < 13 years [16]. In this study, proliferative LN accounted for 97.6% of the cases. We evaluated the renal response rates at 6 and 12 months of induction therapy, with CR rates of 78.7% and 90.9% and PR rates of 19.1% and 9.1%, respectively. Notably, both were higher than previous literature reports, which indicated that only 40–60% of children achieved a CR at 6 months of induction therapy [6]; however, our data showed that 43.3% of children in the traditional treatment group experienced recurrence within 5 years after remission, which is close to the recent data from children with LN in Hong Kong, China (recurrence rate of 41%) [12] and the United States (recurrence rate of 46%) [17]. In this study, 61.9% of relapses occurred after infection, fatigue, or irregular medication use. The 2017 European Children’s LN Evidence-Based Recommendation [18] also indicated that the noncompliance rate in children’s treatment was as high as 50%. Notably, some children with long-term glucocorticoid use experienced different degrees of adverse effects on their psychology, growth, and development due to Cushing syndrome, infection, diabetes, hypertension, and ocular hypertension [19], which led to a decline in compliance with the standard treatment. Therefore, to achieve and maintain sustained renal remission, reduce long-term exposure to glucocorticoids, and improve treatment compliance, it is equally crucial to prevent recurrence and improve prognosis.

Previous studies showed elevated serum BAFF levels are associated with SLE pathogenesis, supporting the basic principle of targeted molecular therapy for SLE. The 2-year BLISS-LN trial [20] showed that belimumab plus standard therapy had a primary efficacy renal response. Yu et al. [21] analyzed the data of the BLISS-LN East Asian subgroup, in which more patients achieved PR (53% vs. 37%; OR, 1.76 [95% CI, 0.88–3.51]) and CR (35% vs. 25%; OR, 1.73 [95% CI, 0.80–3.74]) at week 104 with belimumab treatment, which confirmed that safety and efficacy profiles were consistent with BLISS-LN overall population. Research on BAFF blockade therapy for childhood LN remains limited; however, multiple randomized trials of nonrenal SLE have shown that the efficacy and safety of belimumab are comparable in pediatric and adult patients [22, 23]. This study analyzed the efficacy and safety of the early use of belimumab combined with standard traditional regimens for treating active LN. Forty-seven patients were included in this study. After a median follow-up of 13 months, it was observed that the belimumab group had a higher SLEDAI score (23.59 ± 7.78 vs. 19.13 ± 6.10) at enrollment and a higher median 24-h proteinuria level (75.9 mg/kg. d vs. 63.3 mg/kg. d) and renal pathological activity index (10.47 ± 2.72 vs. 9.59 ± 2.78). Although the statistical results showed no difference in renal remission rate and 1-year recurrence rate between the two groups, the renal remission rate of the belimumab group was slightly higher than that of the traditional treatment group (88.2% vs. 73.3% at months 6 and 92.9% vs. 90.0% at month 12). There was no renal recurrence at 1 year, which was significantly lower than that in the traditional treatment group (13.3%), which indicated that belimumab may effectively reduce disease activity and maintain LN remission without recurrence. However, in this study, the median recurrence time for children in the traditional treatment group was 2 years. In contrast, the follow-up time for the belimumab group was still short, and the number of enrolled cases was not large.

Consequently, whether belimumab can maintain long-term nonrecurrence in patients still requires a larger sample size and extended follow-up time to further observe the long-term efficacy of belimumab. Similarly, our data showed that, under similar conditions of renal remission, glucocorticoid dosage in the belimumab group was significantly lower than that in the traditional treatment group at 6 and 12 months of treatment. This is also consistent with a recent observational study of 17 real-world studies on the application of belimumab to SLE patients, for whom the reduction in SLEDAI score, glucocorticoid equivalent dose, and recurrence rates were significant within 6–12 months of belimumab treatment [24].

Belimumab is a recombinant whole human monoclonal antibody that inhibits the survival of autoreactive B cells and promotes their apoptosis. It has a relatively small impact on advanced B cells, as it can retain a certain level of immunity. In this study, we also compared the serum levels of immunoglobulins, such as IgM, IgG, and IgA, during belimumab therapy. There was a downward trend compared with the baseline; however, no serious adverse reactions were observed clinically. In this multicenter, open-label study on the safety and efficacy of belimumab combined with standard therapy in treating patients with SLE for 13 years [25], the total exposure time of belimumab was 2294 patient-years, with an average median infusion frequency of 115.5 times which showed that with the extension of the observation years, the serum IgG levels of most patients (65.9%) were normal, with only 4.1% of patients experiencing a grade 3 decrease in IgG (250–399 mg/dL) and 2.4% reaching grade 4 (< 250 mg/dL), and there was an average decrease of 16.2% in the IgG levels; however, the risk of infection, including severe infections, did not increase. Therefore, the long-term safety of belimumab was acceptable.

At present, the timing and duration of belimumab treatment in children with LN remain unclear. However, it is unanimously believed that BAFF inhibition is a favorable adjunctive treatment based on the existing traditional treatments for proliferative LN.

This study has the following limitations: single-center design, small sample size, retrospective design, risk of data collection and recording bias, the short application time of belimumab, belimumab not used alone in these patients, and lack of long-term follow-up data.

## Conclusion

As an adjunctive therapy to traditional treatment for children with active LN, belimumab can facilitate an earlier renal response, rapid recovery of serological indicators, faster tapering of glucocorticoid, and with few side effects.

### Supplementary Information

Below is the link to the electronic supplementary material.

Supplementary file1 (PDF 296 KB)

Supplementary file2 (PNG 1422 KB)

## Data Availability

No datasets were generated or analyzed during the current study.

## Abbreviations

BAFF: B cell-activating factor
CKD: Chronic kidney disease
CR: Complete remission
CTX: Cyclophosphamide
ESKD: End-stage kidney disease
Ig: Immunoglobulin
LN: Lupus nephritis
MMF: Mycophenolate mofetil
NR: No renal remission
PR: Partial remission
SLE: Systemic lupus erythematosus
UPCR: Urine protein to creatinine ratio
