# Supplementary material for: Efficacy and safety of belimumab combined with the standard regimen in treating children with lupus nephritis
Source: Eur J Pediatr. 2024 Jun 28;183(9):3987–95. doi: 10.1007/s00431-024-05662-9 (PMC11322259; doi:10.1007/s00431-024-05662-9)
Supplement: Supplementary file 1 — Supplementary file1 (PDF 296 KB) [file 431_2024_5662_MOESM1_ESM.pdf]

Editing Certificate

This document certifies that the manuscript listed below has been edited to ensure language and grammar accuracy and is error free in these aspects. The logical presentation of ideas and the structure of the paper were also checked during the editing process. The edit was performed by professional editors at Editage, a brand of Cactus Communications. The author's core research ideas were not altered in any way during the editing process. The quality of the edit has been guaranteed, with the assumption that our suggested changes have been accepted and the text has not been further altered without the knowledge of our editors.

MANUSCRIPT TITLE

Efficacy and safety of belimumab combined with the standard regimen in treating children with lupus nephritis

AUTHORS

hua rong li

ISSUED ON

June 20, 2024

JOB CODE

LFVZF_3_7

THE EDITAGE Promise QUALITY GUARANTEE

Prabh Grewal
Senior Vice President - Editage

editage helping you get published

Since 2002, Editage has helped over 430,000 authors publish around 1.2 million research papers in scholarly journals across over 1000 disciplines through editorial, translation, transcription, and publication support services. Editage is a brand of Cactus Communications (cactusglobal.com), a science communication and technology company.

CACTUS.

GLOBAL :
+1(833) 979-0061 | request@editage.com

CHINA :
400-120-3020或 021-6020-9400 | fabiao@editage.cn
