# Supplementary figures and images for: Efficacy and safety of belimumab combined with the standard regimen in treating children with lupus nephritis
Source: Eur J Pediatr. 2024 Jun 28;183(9):3987–95. doi: 10.1007/s00431-024-05662-9 (PMC11322259; doi:10.1007/s00431-024-05662-9)

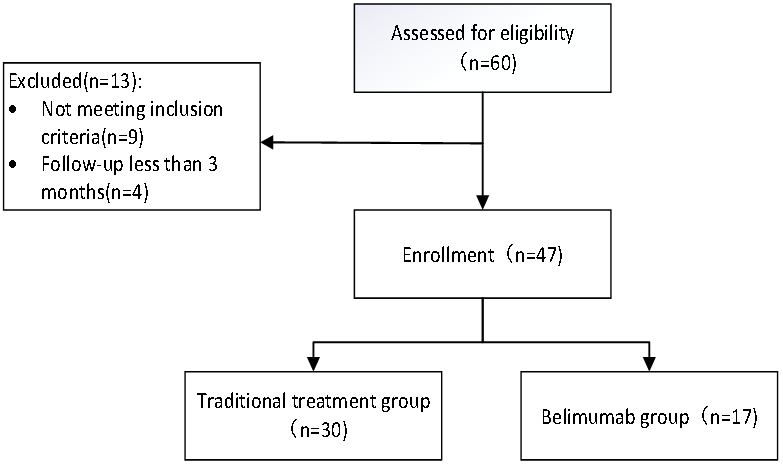

Supplement: Supplementary file 2 — Supplementary file2 (PNG 1422 KB) [file 431_2024_5662_MOESM2_ESM.png]
